# Acinar cell carcinoma of the pancreas: can CT and MR features predict survival?

**DOI:** 10.1186/s40644-025-00859-z

**Published:** 2025-03-21

**Authors:** Monica Cheng, Nikita Consul, Ryan Chung, Carlos Fernandez- del Castillo, Yasmin Hernandez-Barco, Avinash Kambadakone

**Affiliations:** 1 Department of Radiology, Massachusetts General Hospital, Harvard Medical School, 55 Fruit Street, Boston, MA 02114 USA; 2 Department of Radiology, University of Michigan, Ann Arbor, MI USA; 3 Department of Surgery, Massachusetts General Hospital, Harvard Medical School, Boston, MA USA; 4 Department of Gastroenterology, Massachusetts General Hospital, Harvard Medical School, Boston, MA USA

**Keywords:** Pancreas, Acinar cell carcinoma, CT and MRI features, Biomarker

## Abstract

**Objective:**

To evaluate the CT and MRI features of pancreatic acinar cell carcinoma (pACC) and their association with patient outcome and survival.

**Methods:**

This retrospective single-center study included 49 patients with pathology-proven pancreatic acinar cell carcinoma who underwent diagnostic imaging between August 1998 - September 2019. Two radiologists reviewed CT and MRI features independently. Survival was estimated using the Kaplan-Meier method, and Cox proportional-hazards regression model was used to identify factors associated with survival.

**Results:**

pACC tended to present as a solid (31/49, 63.3%) pancreatic head mass (26/49, 53.1%) with ill-defined margins (32/49, 65.3%) and median maximal diameter of 4.1 cm (IQR, 2.9–6.2). Majority of lesions were hypo- or isodense (38/49, 77.6%) compared to normal pancreatic parenchyma, with heterogenous (39/49, 79.6%) enhancement pattern. Biliary ductal dilatation was uncommon, with pancreatic ductal dilatation in 22.4% (11/49) and common bile duct dilatation in 14.3% (7/49). Intralesional calcifications were seen in 6.1% (3/49). Metastasis was present in 71.4% (35/49) of patients at the time of diagnosis. On MRI, 88.9% (16/18) demonstrated diffusion restriction and 59.1% (13/22) with heterogenous enhancement. On multivariate analysis, the imaging presence of T1 hyperintensity (*p* = 0.02), hypoattenuating necrotic components (*p* = 0.02), and splenic vein invasion (*p* = 0.04) were associated with worse survival.

**Conclusion:**

Pancreatic acinar cell carcinoma is a rare pancreatic neoplasm that often presents as a large ill-defined heterogeneously enhancing mass without biliary ductal dilation. T1 hyperintensity, presence of hypoattenuating necrotic components, and splenic vein invasion were independent predictors of survival.

## Introduction

Pancreatic acinar cell carcinoma (pACC) is a rare pancreatic neoplasm, comprising less than 1% of all pancreatic malignancies and associated high mortality with a 5-year survival rate of 17.5% [1, 2]. The exocrine pancreas is largely composed of acinar tissue, but pancreatic neoplasms with acinar differentiation are uncommon compared to other pancreatic neoplasms. Compared to other pancreatic cancers, less is known about pACC in part due to its rarity and nonspecific pattern of disease presentation. Perhaps the most defining albeit uncommon clinical presentation is the lipase hypersecretion syndrome, a paraneoplastic syndrome that manifests as fat necrosis, polyarthralgia, and pancreatitis [3]. However, most patients present with vague clinical symptoms of weight loss, abdominal pain, nausea, and vomiting. Patients with pACC have poor prognosis, although the 5-year survival rate is higher than pancreatic ductal adenocarcinoma [4]. 

Studies investigating radiologic-pathologic features of pACC are limited to small case series and imaging features have not been studied to be associated with prognosis or survival [5–8]. The key imaging features described for pACC include a large well-defined mass without pancreatic ductal dilatation. CT is the most widely utilized imaging modality for initial assessment of pACC, while MRI is useful in equivocal cases or for characterization of incidental liver lesions. While surgical resection is considered first-line treatment, there currently is not a well-established standard of care for patients who present with inoperable disease or for adjuvant therapy [9]. More recently, Sridharan et al. (2021) suggested that surgery was associated with survival benefit among patients who presented with localized disease, while FOLFOX or FOLFIRINOX chemotherapy regimens were associated with improved overall survival in patients presenting with metastatic disease [5]. 

Accurate diagnosis of pACC and identification of prognostic features have the potential to play an essential role in guiding clinical decision making by informing diagnostic workup, staging, and prognosis. In this study, we investigate the CT and MRI features of pancreatic acinar cell carcinoma and their associations with patient outcome and survival.

## Methods

### Patients

This retrospective single-center study was performed after obtaining approval from the institutional review board and was compliant with the Health Insurance Portability and Accountability Act. Our institutional pathology database was searched to identify patients with pathology-proven pACC between August 1998 and September 2019.

**Fig. 1:**
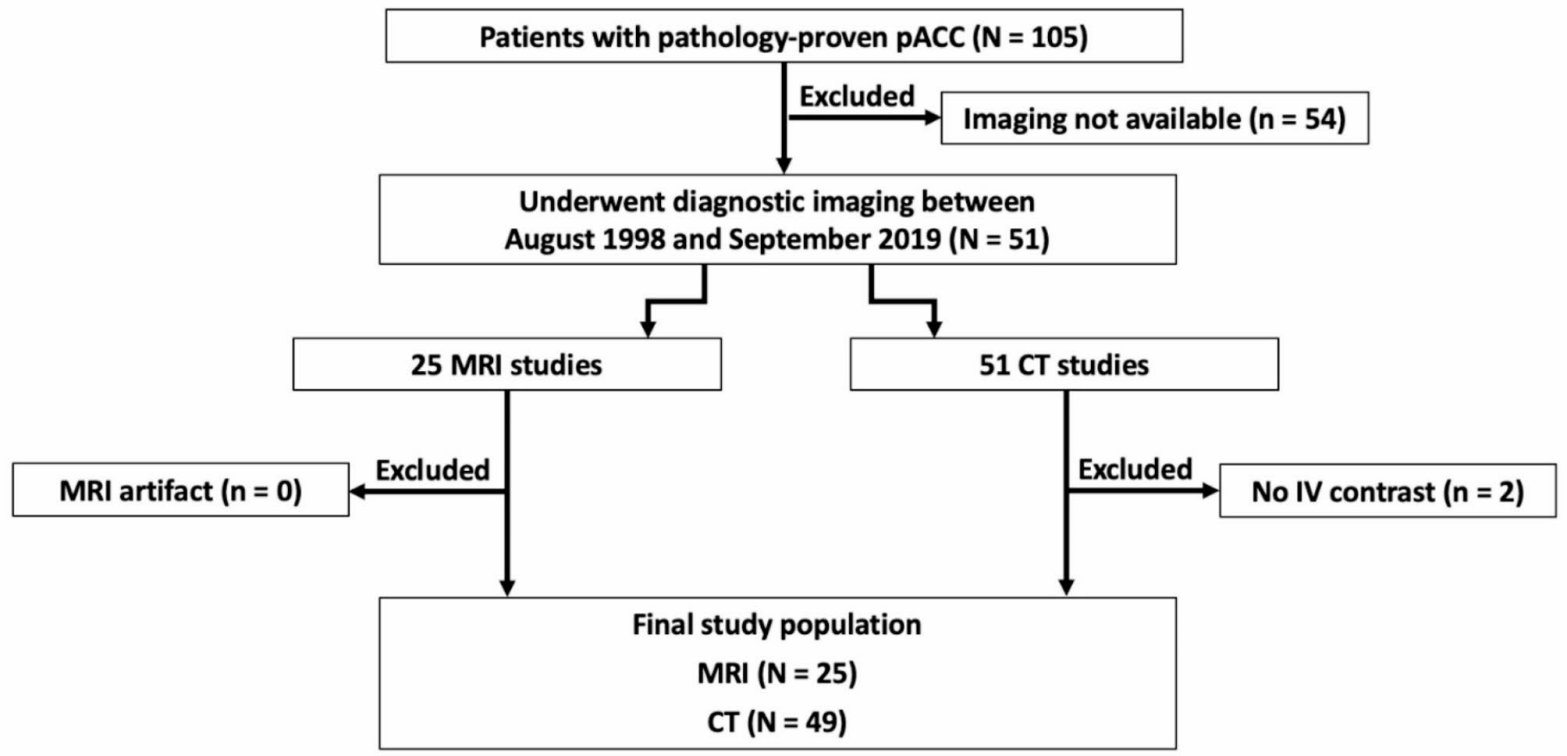
Flowchart of patient inclusion. IV = intravenous; MRI = magnetic resonance imaging; CT = computed tomography; pACC = pancreatic acinar cell carcinoma

Figure 1 illustrates the patient selection process. All patients included in the study had pathology-proven pACC. Exclusion criteria involved imaging performed at outside institutions where studies were unavailable for review, as well as imaging studies conducted without IV contrast, precluding analysis of tumor composition and vascular involvement. The electronic medical records of the patient cohort were assessed by an independent reviewer to obtain patient demographics including age, gender, ethnicity, clinical details, laboratory tests, surgical notes, pathology reports and follow-up details.

### Imaging

All patients included in the study had undergone standard of care CT/MR scanning for evaluation of the pancreatic mass. Imaging protocols are summarized in Table 1. The CT scans were performed on 64-slice, 128-slice and dual energy CT scanners while MRI were performed on 1.5 and 3.0-Tesla scanners. Dynamic contrast enhanced CT was performed after intravenous iodinated contrast media administration with image acquisition in the arterial/pancreatic phase (40 s), portal venous phase (70 s) and delayed phase (3 min). MRI sequences included T1-weighted imaging, T2-weighted imaging, diffusion weighted imaging, and contrast-enhanced imaging.

**Table 1:** CT and MR imaging protocols utilized in this study

Parameter	CT Protocol (*n* = 49)	MRI Protocol (*n* = 25)
**Scanner Type**	64-slice, 128-slice, and dual-energy CT scanners	1.5 and 3.0-Tesla MRI scanners
**Imaging Phases**	Pre-contrast (*n* = 6), Arterial (*n* = 15), Portal Venous (*n* = 46), Delayed (*n* = 11)	Pre-contrast, Arterial, Portal Venous, Delayed
**MRI Sequences**	N/A	T1WI (*n* = 25), T2WI (*n* = 25), DWI (*n* = 19), ADC (*n* = 19), DCE (*n* = 22)

T1WI = T1-weighted imaging; T2WI = T2-weighted imaging, DWI = diffusion-weighted imaging, ADC = apparent diffusion coefficient, DCE = dynamic contrast-enhanced imaging

### Image analysis

All CT and MR examinations were reviewed independently by two radiologists specialized in abdominal imaging (one reader with 3 years of post-fellowship experience and one abdominal radiology fellow) on PACS workstations. The independent radiology readers were aware of pACC diagnosis but were blinded to all other clinical and pathological information. Any subsequent discordances were adjudicated by a third radiologist with 14 years of abdominal subspecialty experience.

The readers evaluated the CT/MR images using a predesigned template created to evaluate tumor characteristics, nodal involvement, and metastatic disease. The images were assessed for tumor size, location within the pancreas, composition, margination, presence of calcifications, lymphadenopathy, metastasis, biliary ductal dilatation, pancreatic parenchymal atrophy, enhancement pattern, and vascular involvement. Margination was categorized as well-defined or ill-defined, where ill-defined margins was defined as having indistinct borders. Tumor size was measured in three dimensions—transverse, anterior-posterior, and cranio-caudal. Location within the pancreas was defined as uncinate process, neck, head, body, and tail. Internal density of the lesion was described as hypo-, iso-, or hyperattenuating compared to the surrounding pancreatic parenchyma; on CT, if areas of low attenuation were seen, the tumor was classified as necrotic in correlation with pathologic assessment [10–12]. Enhancement pattern on both CT and MRI was described as hypo- or hyperenhancing compared to surrounding parenchyma, on arterial, portal venous, and delayed phase imaging. The tumors were categorized as homogenous versus heterogenous, based on visual assessment. Vascular involvement on CT and MRI was stratified by no involvement, abutment (less than 180-degree contact), encasement (greater than 180-degree contact), or thrombosis based on the National Comprehensive Cancer Network guidelines for pancreatic ductal adenocarcinoma (PDAC). Signal intensity characteristics of the tumor on T1-weighted imaging, T2-weighted imaging, and diffusion weighted imaging were categorized as hyper-, iso-, or hypointense based on the predominant component of the lesion relative to the normal pancreatic parenchyma. Diffusion restriction was defined as hyperintensity on diffusion weighted imaging with corresponding hypointensity on apparent diffusion coefficient. Lymphadenopathy was considered for short axis diameter greater than 1 cm. Metastatic sites including the lung, liver, or bone were assessed.

### Reference standard and follow-up

The following information was obtained from the electronic medical records: age, gender, ethnicity, smoking status, baseline metastatic status, and details of pathological specimens (e.g., T stage, treatments received, follow-up). Lymph node involvement on imaging was correlated with pathologic assessment.

### Statistical analysis

Interobserver agreement between the two radiologists was assessed with intra-class correlation coefficients (ICC) and Cohen’s Kappa (*k*) for continuous and categorical variables, with degree of agreement classified as: 0.01–0.20, poor, 0.21–0.40, fair, 0.41–0.60, moderate, 0.61–0.80, substantial, and 0.81–1.00, excellent [13–15]. The consensus imaging interpretation after adjudication by the third radiologist was used for the rest of the analyses. Kaplan-Meier analysis was used to calculate survival, defined as the time from diagnostic imaging to death or last follow-up. Univariate and multivariate Cox proportional-hazards regression model was used to identify clinical and imaging variables that were independently associated with survival. A p-value less than 0.05 was regarded as statistically significant. R (version 4.3.1) was used for statistical analysis.

## Results

### Patient and clinicopathological characteristics

Table 2 describes the patient characteristics. Based on the inclusion and exclusion criteria, 49 patients were identified for analysis who underwent a total of 74 diagnostic imaging studies between August 1998 and September 2019. This included 49 CT and 25 MRI examinations within the same diagnostic period, of which 25 patients underwent both CT and MRI. Initial pathologic diagnosis was made based on biopsy sample in 37 patients and surgical resection in 12 patients. Of the 49 patients, 36 [73.5%] were males and 13 [26.5%] females with a median age of 66 years (interquartile range [IQR] 58–75). Most patients were white (*n* = 45 [91.8%]) and were former or active smokers (*n* = 27 [55.1%]). Thirty-five patients (71.4%) presented with metastases at the time of diagnosis, most commonly in the liver (*n* = 23 [65.7%]). Patients underwent Whipple (*n* = 12 [24.5%]), distal pancreatectomy (*n* = 13 [26.5%]), or no surgery (*n* = 24 [49.0%]), and most tumors were ≥ pT3 (*n* = 17 [60.7%]) based on the 8th edition of the American Joint Commission on Cancer TNM staging system [16]. Thirty-five patients (71.4%) received systemic treatment in the form of chemotherapy and/or radiation therapy. Metastatic pathology-proven lymph nodes were present in 27 patients (55.1%).

**Table 2:** Clinicopathological and imaging characteristics of patients with pancreatic acinar cell carcinoma

Characteristics	Category	No. of patients (%)*
Clinical findings		
Age (years)		66 (58, 75)*
Gender	Male	36 (73.5)
	Female	13 (26.5)
Ethnicity	White	45 (91.9)
	Black	1 (2.0)
	Asian	2 (4.1)
	Other	1 (2.0)
Baseline metastatic status	Loco-regional	14 (28.6)
	Metastatic	35 (71.4)
Surgery type	Whipple	12 (24.5)
	Distal pancreatectomy	13 (26.5)
Systemic treatment	None	14 (28.6)
	Neoadjuvant only	12 (24.5)
	Neoadjuvant and adjuvant	6 (12.2)
	Adjuvant	17 (34.7)
Pathological tumor stage	pT1	5 (17.9)
	pT2	6 (21.4)
	pT3	14 (50.0)
	pT4	3 (10.7)
Metastatic lymph nodes on pathology	Present	12 (42.9)
	Absent	16 (57.1)
CT findings (*n* = 49)		
Median size on CT (cm)		4.1 (2.9, 6.2)*
Margination	Well-defined	17 (34.7)
	Ill-defined	32 (65.3)
Hypoattenuating areas	Present	19 (38.8)
	Absent	30 (61.2)
Lymphadenopathy	Present	27 (55.1)
	Absent	22 (44.9)
Vascular thrombus	Present	4 (8.2)
	Absent	45 (91.8)
Biliary ductal dilatation	Pancreatic duct	11 (22.4)
	Common bile duct	7 (14.3)
Splenic vein involvement	Abutment	12 (24.5)
	Encasement	14 (28.6)
	No involvement	23 (46.9)
Parenchymal atrophy	Present	7 (14.3)
	Absent	42 (85.7)
Calcifications	Present	3 (6.1)
	Absent	46 (93.9)
MRI findings (*n* = 25)		
Diffusion restriction (*n* = 18)	Present	16 (88.9)
	Absent	2 (11.1)
T1WI	Hyperintense	5 (20.0)
	Hypointense	10 (40.0)
	Isointense	10 (40.0)
T2WI	Hyperintense	17 (68.0)
	Hypointense	2 (8.0)
	Isointense	6 (24.0)

* Presented as median and interquartile range; others are presented as number of patients and percentages

### Imaging findings

The most common presentation of pACC was an enhancing solid mass (63.3%, *n* = 31) in the pancreatic head (53.1%, *n* = 26). The average maximal diameter was 5.0 cm while the median maximal diameter was 4.1 cm (IQR, 2.9–6.2). Thirty-two (65.3%) demonstrated ill-defined margins, 38.8% (19/49) contained hypoattenuating components corresponding to pathology-proven necrosis, 14.3% (7/49) showed upstream pancreatic parenchymal atrophy, and 6.1% (3/49) had calcifications. Upstream pancreatic ductal dilatation was seen in 22.4% (11/49) of cases with the main duct dimension measuring up to 18 mm, and 14.3% (7/49) had common bile ductal dilatation measuring up to 20 mm. Vascular thrombosis was present in 4 (8.2%) patients.

All lesions demonstrated contrast enhancement on CT and MRI. On portal venous phase (*n* = 46), the majority were hypo- or isoattenuating (82.6%, *n* = 38) compared to normal pancreatic parenchyma and tended to have heterogenous (39/49, 79.6%) enhancement pattern. On delayed phase imaging (*n* = 11), all hypo- (1/11) or isoattenuating (6/11) lesions were unchanged from portal venous phase. There were two isoattenuating lesions on portal venous phase that became hyperenhancing on delayed phase imaging. On arterial phase imaging (*n* = 15), 13 demonstrated consistent attenuation pattern across portal venous and/or delayed phase imaging. One hypoattenuating lesion became isoattenuating on delayed phase imaging, and another isoattenuating lesion became hyperattenuating on delayed phase imaging. On MRI, 89.4% (17/19) demonstrated diffusion restriction. Most lesions (80%, 20/25) were T1 hypo- or iso-intense, and 68% (17/25) were T2 hyperintense. 59.1% (13/22) showed heterogenous enhancement on MRI. The inter-reader agreement between the two radiologists was excellent for MRI characteristics on T1, T2, and diffusion weighted imaging (*k* = 0.813). Agreement was substantial for splenic vein involvement (*k* = 0.70, 95% CI 0.53–0.86), moderate for assessment of thrombosis (*k* = 0.52, 95% CI 0.07–0.97), and fair for enhancement pattern (*k* = 0.35, 95% CI 0.11–0.6).

### Follow-up and survival

**Fig. 2:**
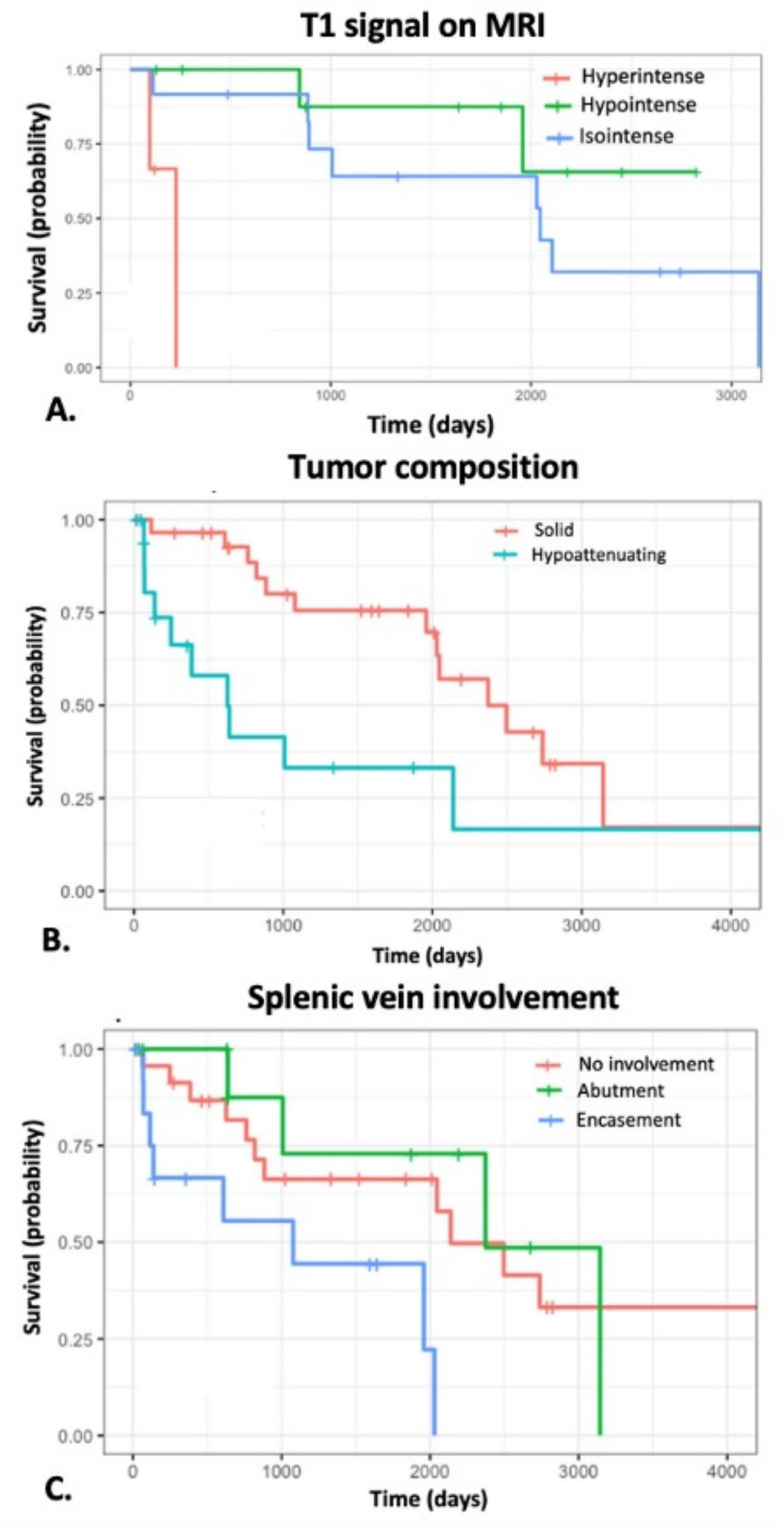
Kaplan-Meier survival curves stratified to CT and MRI findings. (**A**) T1 hyperintensity on MRI, (**B**) presence of hypoattenuating components on CT, and (**C**) splenic vein encasement were associated with worse survival

**Fig. 3:**
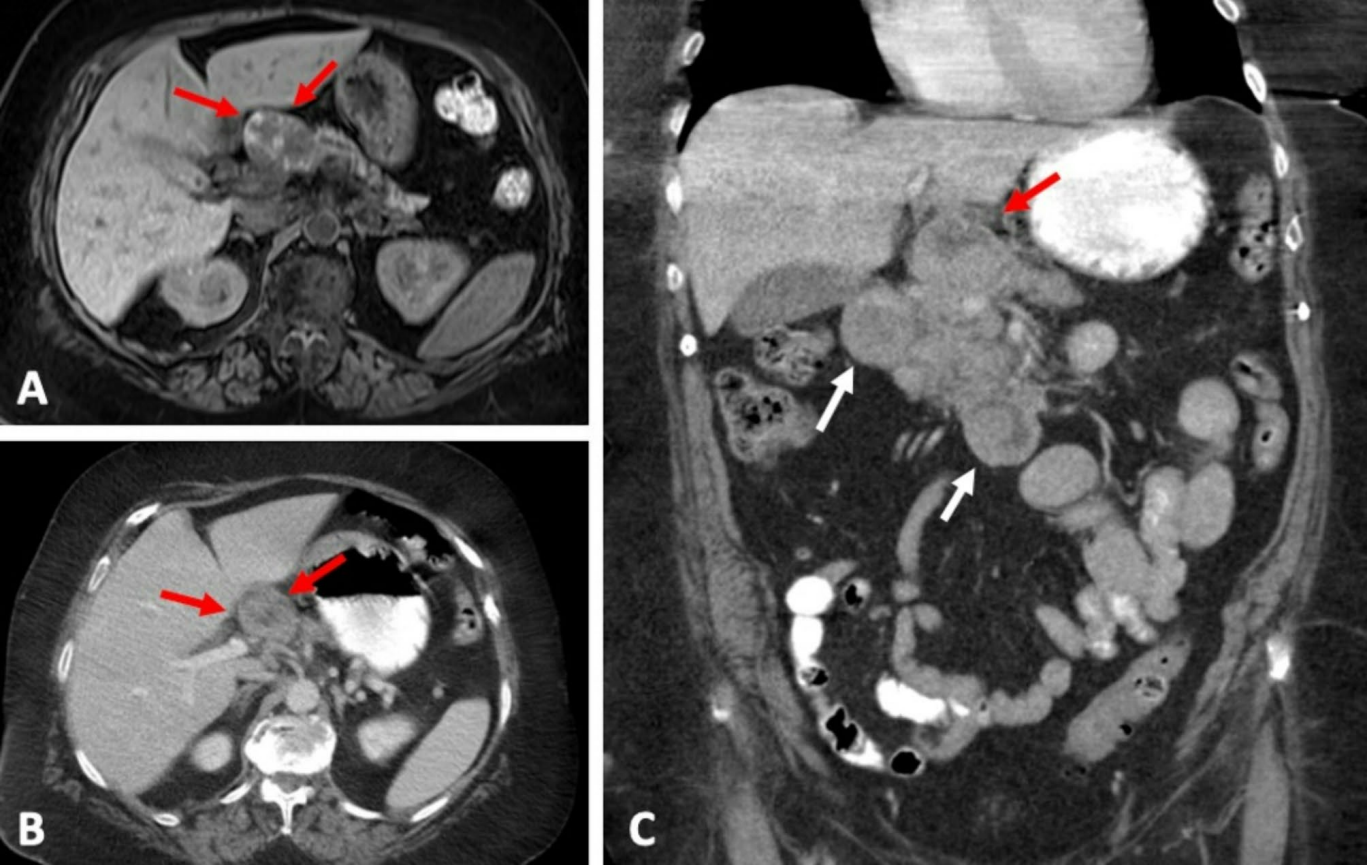
78-year-old female with acinar cell carcinoma in the pancreatic head (red arrows) demonstrating hyperintensity on axial T1-weighted MR image (**A**) and hypoattenuating components on CT (**B**, **C**). Locoregional metastasis to the peripancreatic lymph nodes (**C**) (white arrows) was present at the time of diagnosis. The patient died 248 days after diagnosis

**Fig. 4:**
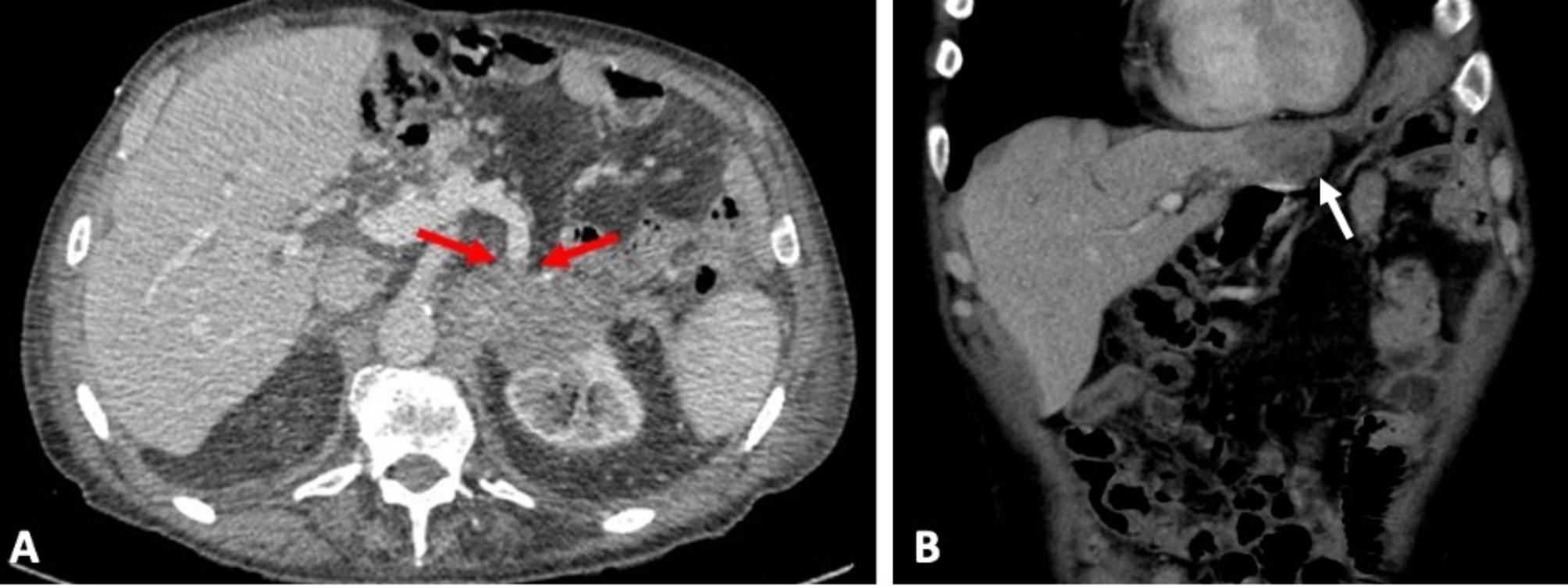
80-year-old male with acinar cell carcinoma of the pancreatic tail demonstrating splenic vein encasement (**A**) (red arrows) and with liver metastasis (**B**) (white arrow). Patient died 138 days after diagnosis

Twenty-four (47.1%) patients died after a median follow-up time of 853 days (IQR 352–2069 days). Survival curves stratified by imaging variables are shown in Fig. 2. On multivariate analysis controlling for other variables (Table 2), the imaging presence of T1 hyperintensity (*p* = 0.02), hypoattenuating necrotic components on CT (*p* = 0.02), and splenic vein invasion (*p* = 0.04) were also associated with worse survival (Fig. 2). Figure 3 illustrates the imaging features of T1 hyperintensity on MRI and hypoattenuating necrotic components on CT, and Fig. 4 demonstrates splenic vein invasion.

**Fig. 5:**
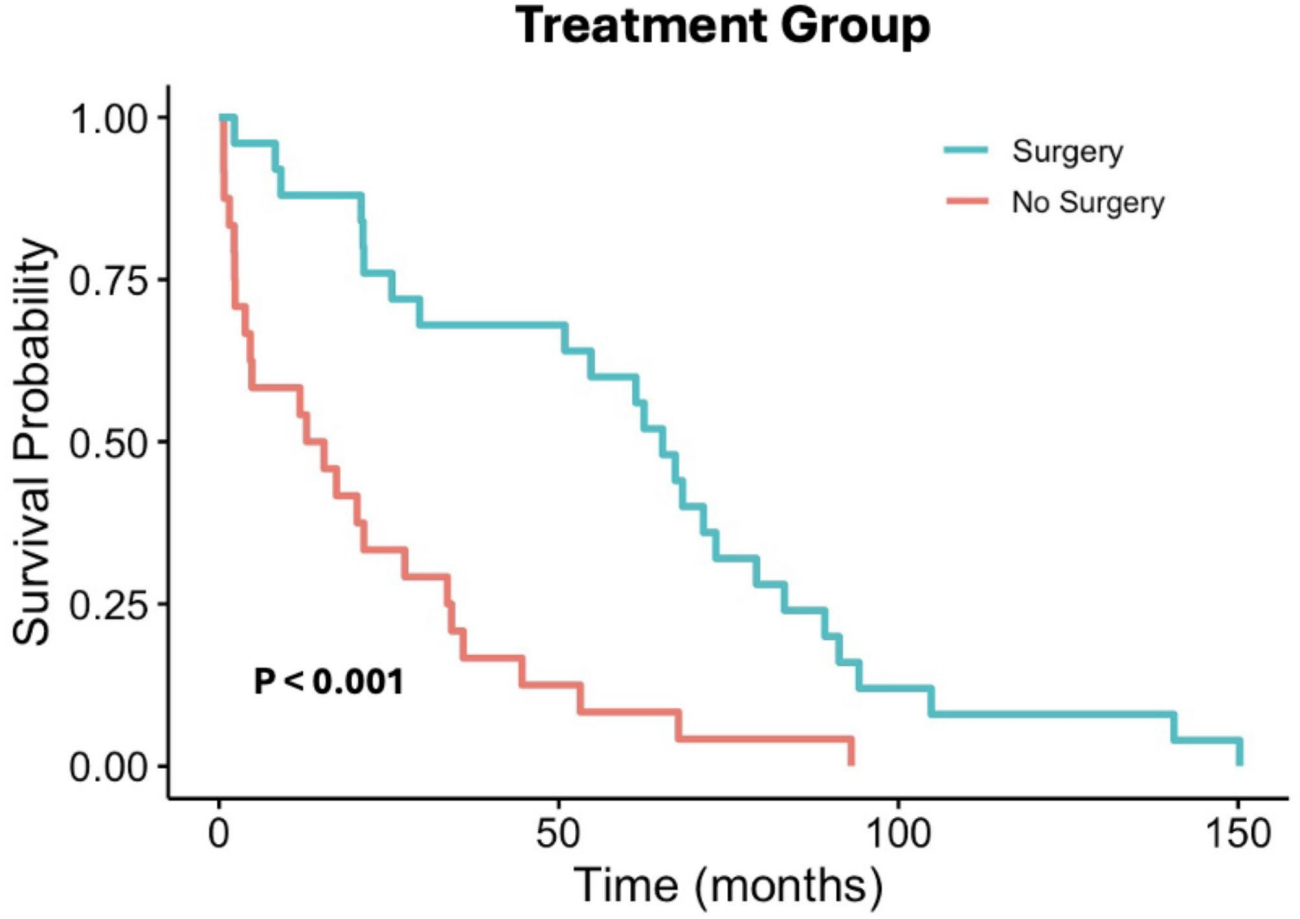
Kaplan-Meier survival curve stratified to treatment group, based on whether patients underwent surgical resection of the primary tumor or non-surgical management. Primary surgical resection of pACC was associated with improved survival (*p* < 0.001)

Older age (*p* = 0.005) and higher pathologic stage (*p* = 0.04) were significantly associated with worse survival, with older age being significant on multivariate analysis (*p* = 0.02). Surgical resection of the primary tumor was associated with improved survival, compared to non-surgical management (*p* < 0.001) (Fig. 5). The 5-year survival rate for patients who underwent surgery was 60.0%, as compared to 8.3% for those who underwent non-surgical management. Other clinicopathologic factors were not associated with survival.

## Discussion

Pancreatic acinar cell carcinoma is a rare entity with heterogenous clinicopathological spectrum of disease which impacts therapeutic management and patient outcomes [17]. Imaging diagnosis of pACC is challenging due to limited literature and in this study we investigated the role of imaging as a biomarker for predicting outcome and association with survival.

We found that a majority of pACC were large solid masses in the pancreatic head with ill-defined margins. pACC tends to present at a larger size at the time of diagnosis compared to PDAC, with a reported average size ranging from 4 to 10 cm [6, 8, 18]. In our study, the average maximal diameter was 5.0 cm while the median maximal diameter was 4.1 cm. Most (53.1%) presented in the pancreatic head and 71.4% were metastatic at presentation, in keeping with prior studies [6, 19, 20]. Biliary ductal dilatation was uncommon, with only 22.4% demonstrating pancreatic ductal dilatation, helping to distinguish from other entities such as PDAC in which obstructive biliary dilatation is more common and present in up to 90% of cases [21]. Bhosale et al. reports calcifications in 6% of tumors, in keeping with our study results in which 3/49 (6.1%) demonstrated calcifications. The relative absence of calcifications in pACC can help distinguish it from tumors where calcifications are more frequently associated with the tumor such as pancreatic neuroendocrine tumors, and cystic neoplasms including solid pseudopapillary neoplasm, serous cystic neoplasm, and mucinous cystic neoplasm [22]. 

All lesions demonstrated contrast enhancement on CT and MRI. On portal venous phase of CT, the majority were hypo- or isodense (82.6%) to the normal pancreatic parenchyma, thought to reflect the lesion’s hypovascularity, and tended to have a heterogenous enhancement pattern (79.6%). These findings affirm those of previously reported limited patient series [6, 8]. Hence, pACC should be considered in the differential diagnosis for large heterogeneously enhancing hypodense pancreatic masses in the absence of biliary or pancreatic ductal dilatation [7, 8, 19, 23]. While pACC and PDAC share imaging features, key distinctions exist. pACC typically appears as a large heterogenous mass without significant biliary dilatation, whereas PDAC often presents with biliary obstruction. Both demonstrate diffusion restriction and unlike previous studies, the present study found that pACC often to have ill-defined margins resembling PDAC.

Pancreatic neuroendocrine tumor may be another differential consideration on pathology for these pancreatic neoplasms, and imaging may be able to help in recognizing certain distinguishing features. Well-differentiated pancreatic neuroendocrine tumors (grade 1 and 2) are typically small well-defined lesions with intense homogenous enhancement [24]. This appearance contrasts with the large ill-defined heterogeneously enhancing imaging presentation of pACC, although high grade pancreatic neuroendocrine tumors may potentially demonstrate greater degree of inhomogeneity and heterogenous enhancement pattern. On MRI, case studies and limited case series have suggested that pACC shows diffusion restriction and heterogenous enhancement pattern [25, 26]. These findings are affirmed in our study in which 89.4% demonstrated diffusion restriction, and 59.1% with heterogenous enhancement.

Approximately 50% of the cohort died after a median follow up time of 853 days. According to small-cohort clinicopathologic analysis performed in 1992, age and pathologic stage were associated with survival in which patients who presented before the age of 60 survived twice as long as older patients [27]. Indeed, our study affirmed that location within the pancreas did not demonstrate significant correlation with survival, and higher pathologic stage and older age were associated with worse survival, with older age being significant on multivariate analysis. Treatment modality was also associated with survival, in which surgical resection of the primary tumor was associated with improved survival compared to non-surgical management.

We found that a few imaging features were associated with survival. T1 hyperintensity within the tumor on MRI was associated with worse survival; given our knowledge of which materials have intrinsic T1-shortening properties, the presence of T1 hyperintense signal in the pancreatic mass may relate to intralesional hemorrhage, necrosis with proteinaceous debris, or the presence of intravascular thrombi. Multivariate analysis demonstrated that the imaging presence of hypoattenuating components corresponding to pathology-proven necrotic contents were associated with worse survival. These results align with recent studies indicating tumor necrosis on imaging may be predictive of tumor aggressiveness in other pancreatic tumors including PDAC; Anderson et al. (2023) notes association of MRI-evident necrosis with larger size PDAC tumors as well as higher likelihood of regional lymphadenopathy and metastases [28, 29]. Previous literature supports the finding in which hypoattenuating pancreatic cancers tended to correlate with necrosis and dense fibrous stroma from desmoplastic reaction, which portend poorer prognosis [10, 30]. Notably, our study found that encasement of the splenic vein was associated with worse survival compared to no involvement. These findings represent an extension of previous literature illustrating poor prognosis of pancreatic cancers with splenic vessel involvement. Crippa et al. (2018) had demonstrated in a meta-analysis of patients with PDAC involving the pancreatic body and/or tail that splenic vessel involvement was associated with worse survival, reflecting stigmata of more aggressive disease [31]. Recognizing the clinical implications of imaging features such as T1 hyperintensity, hypoattenuating necrosis, and vascular invasion is crucial, as their correlation with tumor aggressiveness may influence treatment decision-making. These features have the potential to serve as imaging biomarkers for risk stratification, facilitating therapeutic optimization, prognostic assessment, and treatment selection.

Compared to previous studies on pACC, our study contributes to the growing knowledge of imaging characterization on both CT and MRI while also providing novel insights by correlating imaging features with survival outcomes, an aspect not previously investigated in the literature. Prior studies with limited sample sizes have included a maximum of 30 patients, with most cohorts comprising fewer than 20 individuals [6–8]. These studies primarily investigated the CT characteristics of pancreatic acinar cell carcinoma (pACC), with Tatli et al. (2005) being the only study to evaluate MRI features, albeit in just two patients [6]. The variability in study methodologies is likely attributable to the small sample sizes inherent to this rare malignancy. Patient demographics were largely consistent across studies, with a mean age in the 60s and a predominance of male patients. The overall CT features of pACC seen in the present study are consistent with previous studies, confirming that pACC typically presents as a large, heterogeneous mass, with biliary ductal dilatation and calcifications being uncommon features.

This was a retrospective single-institution study with its inherent limitations related to such a study design. Further validation of these findings in a multi-center or larger cohort would be beneficial. Assessment of prognostic features was limited for patients whose care was transferred to outside institutions, in which subsequent records were unavailable for review. The number of lesions demonstrating T1 hyperintensity and splenic vein involvement represented the relatively smaller but significant proportion of patients who may have worse prognosis. Due to the long inclusion period required for the identification of this exceptionally rare pancreatic neoplasm, the total number of available MRI studies for analysis was limited. Furthermore, some MR examinations lacked complete data for the assessment of parameters such as diffusion-weighted imaging and dynamic contrast enhancement patterns. Variability in the imaging phases of contrast-enhanced CT limited the ability to perform a comprehensive analysis of temporal enhancement patterns. For patients who underwent biopsy only without surgical resection, radiologic-pathologic correlation was not feasible. Nonetheless, the present study offers valuable new information to bridge our understanding of this rare disease entity.

In conclusion, pancreatic acinar cell carcinoma often presents as a large ill-defined mass without biliary ductal dilation. T1 hyperintensity, presence of hypoattenuating necrotic components, and splenic vein invasion may serve as independent predictors of survival, and our results contribute to the growing body of knowledge on the diagnostic and prognostic imaging features of this rare pancreatic malignancy.

## Data Availability

The datasets generated during and/or analyzed are available from the corresponding author upon request.
